# Combined intravitreal injection of bevacizumab and a ROCK inhibitor (fasudil) for refractory macular edema secondary to retinal vein occlusion: a pilot study

**DOI:** 10.1186/s40942-022-00389-x

**Published:** 2022-06-11

**Authors:** Sahba Fekri, Ramin Nourinia, Babak Rahimi-Ardabili, Arash Daneshtalab, Hamideh Sabbaghi, Hamid Ahmadieh, Bahareh Kheiri

**Affiliations:** 1 Ophthalmic Research Center, Research Institute for Ophthalmology and Vision Science, Shahid Beheshti University of Medical Sciences, No 23, Paidarfard St., Boostan 9 St., Pasdaran Ave, Tehran, 16666 Iran; 2 Department of Ophthalmology, Labbafinejad Medical Center, Shahid Beheshti University of Medical Sciences, Tehran, Iran; 3 Ophthalmic Epidemiology Research Center, Research Institute for Ophthalmology and Vision Science, Shahid Beheshti University of Medical Sciences, Tehran, Iran; 4 Department of Optometry, School of Rehabilitation, Shahid Beheshti University of Medical Sciences, Tehran, Iran

**Keywords:** Bevacizumab, Fasudil, Macular edema, Retinal vein occlusion, ROCK inhibitor

## Abstract

**Background:**

To investigate the adjunctive effect of an intravitreal ROCK inhibitor (fasudil) in combination with intravitreal bevacizumab (IVB) on refractory macular edema secondary to retinal vein occlusion (RVO).

**Methods:**

In this prospective interventional case series, 17 eyes of 17 patients (10 men, 7 women) with refractory RVO-related macular edema underwent three consecutive intravitreal injections of bevacizumab plus fasudil. Monthly evaluation was continued up to 12 months and IVB injection was performed if needed during the follow-up. Changes in the best corrected visual acuity (BCVA) was the primary outcome measure. The secondary outcome measures included central macular thickness (CMT) changes and any adverse events.

**Results:**

BCVA significantly improved (mean change: −0.15 LogMAR; P = 0.017) after 3 consecutive intravitreal injections of fasudil in combination with bevacizumab. CMT significantly decreased (mean change: −206 µm; P = 0.028). The anatomical and functional improvement was maintained during the 12 month follow-up. No adverse effects were noticed.

**Conclusion:**

Intravitreal ROCK inhibitors may break the resistance to anti-VEGF therapy and improve the RVO induced macular edema via affecting the VEGF-independent pathways.

## Background

Retinal vein occlusions (RVO), including central retinal vein occlusion (CRVO) and branch retinal vein occlusion (BRVO), are the second most common cause of retinal vascular disorders leading to profound visual impairment in many patients [1]. The most common cause of visual loss in patients with RVO is macular edema (ME). Currently, the first line treatment for RVO-related ME is intravitreal injection of anti-vascular endothelial growth factors (anti-VEGF) drugs [2]. Although the therapeutic response may be favorable in most patients at the beginning of the therapy, some may experience recurrence of ME or show unresponsiveness to anti-VEGF intravitreal injections. Shifting to other types of anti-VEGF drugs or intravitreal steroids are available choices, but treatment failure, increased rate of complications and cost may have adverse effects on the outcomes. This necessitates a more effective and safer adjuvant treatment for chronic/recurrent and refractory cases. The Rho/ROCK signaling pathway controls various functions such as cell adhesion, contraction, migration, proliferation, apoptosis, angiogenesis, chemotaxis and vasodilation, and may provide neural protection [3]. This wide range of activity causes ROCK inhibitors to be new treatment options for diverse ocular disorders including corneal epithelial and endothelial damage, glaucoma, diabetic macular edema, retinal and choroidal neovascularization, and optic nerve disorders [3]. To our best knowledge, this is the first study evaluating the adjunctive role of a ROCK inhibitor (fasudil) in the management of refractory ME secondary to RVO.

## Materials and methods

In this prospective interventional case series, patients with the history of refractory macular edema due to retinal vein occlusion were recruited in the study. Inclusion criteria were as follows: (1) central macular thickness (CMT) ≥ 250 µm, (2) best corrected visual acuity (BCVA) ≤ 20/40, (3) history of at least 3 intravitreal injections of bevacizumab (IVB), and (4) at least 1 month interval with the last injection of IVB. Patients with uncontrolled blood pressure, renal failure, diabetic retinopathy, severe anemia, corneal opacity, cataract, glaucoma, history of uveitis, history of vitreoretinal surgery, history of cataract surgery within the past 6 months, and any macular disorder other than RVO-related macular edema were excluded from the study. Cases of ischemic type CRVO and non-perfused BRVO were not included in this study. Refractory macular edema was defined as CMT ≥ 250 µm with less than 10% reduction after at least 3 consecutive IVB injections. This study was approved by the Ethics Committee of the Ophthalmic Research Center at Shahid Beheshti University of Medical Sciences. The study was conducted according to the tenets of the Declaration of Helsinki and informed consent was obtained from all patients.

Comprehensive ophthalmic examination including BCVA assessment, anterior segment evaluation, applanation tonometry, dilated funduscopy, and spectral-domain optical coherence tomography (Heidelberg Engineering, Heidelberg, Germany) were performed for all patients at baseline and at each follow-up visit. Fluorescein angiography (FAG, Heidelberg Engineering, Heidelberg, Germany) was performed at baseline. Each patient received 3 consecutive intravitreal injections of 50µMolar/L (0.025 mg/0.05 mL) fasudil [IVF, (Asahi Kasei Pharma, Tokyo, Japan)] [4–6] plus 1.25 mg/0.05 mL bevacizumab (Avastin, made for F. Hoffmann-La Roche, Basel, Switzerland) with two different syringes via separate routes, followed by anterior chamber paracentesis. Combined injections were performed at one month intervals. Patients were followed for 12 months and treatment was continued with IVB injections if needed.

## Statistical analysis

To describe the data, frequency (percent), mean ± SD, median, and range were used. To evaluate the changes within groups during the follow-up, paired t-test was done. All statistical analyses were performed by SPSS software (IBM Corp. Released 2017. IBM SPSS Statistics for Windows, Version 25.0. Armonk, NY: IBM Corp.)

## Results

Seventeen eyes of 17 patients (10 men, 7 women) with refractory RVO-related macular edema were included in this study. There was no missed data for any of the patients during the follow up period. The mean age was 60.14 ± 6.36 years (range, 50 to 74). Of these, 10 had the history of BRVO and 7 patients were CRVO cases. Median number of previous bevacizumab injections was 7 (range, 3 to 18). Baseline characteristics of patients is shown in Table 1. The interval between the last injection of bevacizumab and entering the study ranged from 2 to 13 months. Mean BCVA was 0.66 ± 0.47 LogMAR at baseline which was significantly improved to 0.51 ± 0.33 LogMAR at month 3, one month after the last injection of IVB/IVF (P = 0.017). For CMT, the corresponding figures were 607 ± 271 µm and 404 ± 185 µm (P = 0.028). The changes in CMT and BCVA during follow-up time points are shown in Tables 2 and 3, respectively. CMT reduction sustained up to month 12 and was significantly reduced in comparison with baseline (P < 0.05), but the changes in CMT at follow-up intervals (3, 6, 9, and 12 months) was not significant. Although BCVA improved during follow-up, the change was not statistically significant at month 6 compared with baseline (Table 3). No intraocular pressure (IOP) rise or any adverse effects were observed.

**Table 1:** Baseline characteristics of patients

Parameter	Total (%)
Sex	17
Male	10 (58.8%)
Female	7 (41.2%)
Age (years)	
Mean ± SD	60.14 ± 6.36
Eye	
OD	8 (47.1%)
OS	9 (52.9%)
RVO type	
BRVO	10 (58.8%)
CRVO	7 (41.2%)
Time to injection (months)^a^	
Median (range)	4.5 (2–13)
Previous bevacizumab injections (n)	
Median (range)	7 (3–18)

^a^The interval between the last injection of bevacizumab and entering to the study

**Table 2:** Central macular thickness changes compared with baseline during follow-up

		Mean ± SD	Median (range)
Baseline (µm)	CMT	607 ± 271	643 (257–1166)
Month 3	CMT	404 ± 185	315 (234,882)
	Change	−206 ± 299	−93 (−856–129)
	P-value within	0.028	
Month 6	CMT	412 ± 146	361 (188,698)
	Change	−183 ± 226	−148 (−637,228)
	P-value within	0.007	
Month 9	CMT	414 ± 154	420 (233,810)
	Change	−209 ± 249	−220 (−698,144)
	P-value within	0.01	
Month 12	CMT	379 ± 125	404 (200,605)
	Change	−223 ± 227	−149 (−581,57)
	P-value within	0.004	

*CMT* central macular thickness

**Table 3:** Best corrected visual acuity changes compared with baseline during follow-up

		Mean ± SD	Median (range)
Baseline	LogMAR	0.66 ± 0.47	0.6 (0,1.79)
Month 3	LogMAR	0.51 ± 0.33	0.4 (0,1.39)
	Change	−0.15 ± 0.23	−0.1 (−0.61,0.20)
	P-value within	0.017	
Month 6	LogMAR	0.57 ± 0.37	0.5 (0,1.48)
	Change	−0.09 ± 0.29	0 (−0.61,0.60)
	P-value within	0.223	
Month 9	LogMAR	0.5 ± 0.38	0.4 (−0.12,1.48)
	Change	−0.16 ± 0.25	−0.18 (−0.61,0.30)
	P-value within	0.023	
Month 12	LogMAR	0.52 ± 0.38	0.4 (−0.12,1.48)
	Change	−0.16 ± 0.22	−0.12 (−0.61,0.40)
	P-value within	0.016	

*LogMAR* logarithm of minimum angle of resolution

## Discussion

This pilot study revealed that addition of intravitreal fasudil to bevacizumab could break resistance to anti-VEGF therapy in eyes with chronic/recurrent and refractory RVO-related ME and led to significant anatomical and functional improvement. Moreover, the beneficial effects of combination therapy persisted in spite of the discontinuation of fasudil injections. Management of macular edema is one of the most important challenges in patients with RVO. Activation of multiple pathways following RVO leads to macular edema. VEGF plays the most important role in this process; therefore anti-VEGF drugs are the mainstay of treatment for RVO-related ME. However, the diversity of mechanisms involved in the pathogenesis of RVO-related macular edema led us to investigate the possible effects of the drugs affecting other pathways in eyes that were not responsive to multiple injections of intravitreal bevacizumab. Rho is a small GTPase that can be activated by guanine nucleotide exchange factors (GEF). GTP-bound Rho-A subsequently activates ROCKs to phosphorylate a variety of substrates. Two isoforms, ROCK-I and ROCK-II, have been found to be present in the cornea, trabecular meshwork, iris, optic nerve and retina [7]. Indeed, retina is a proper target tissue for ROCK inhibitor drugs. Mechanisms of action for ROCK inhibitors in the treatment of retinal vascular disorders include suppression of intercellular adhesion molecule-1 (ICAM-1) expression and leucocyte adhesion to vascular endothelium, inhibition of vasoconstriction and endothelial cell apoptosis, reduction of vessel permeability, increasing blood flow, and improving retinal perfusion [8–14]. Moreover, intravitreal injection of ROCK inhibitors can even decrease the VEGF level [11].

There are multiple experimental and human studies focusing on the role of ROCK inhibitors including fasudil, ripasudil, and netarsudil in the management of various ocular disorders. In a randomized clinical trial, Ahmadieh et al. compared the efficacy of combination therapy (bevacizumab + fasudil) versus monotherapy (bevacizumab only) in the treatment of refractory diabetic macular edema (DME) [6]. They reported anatomical and functional improvement in both groups but it was significantly more prominent in the combined group. Interestingly, after the cessation of injections, BCVA and CMT returned to the baseline in the monotherapy group, whereas the therapeutic effects persisted up to 6 months in the combined group. They concluded that adjunctive intravitreal injection of a Rho-kinase inhibitor may enhance and prolong the therapeutic effects of anti-VEGF drugs for center-involving DME. Our findings showed that similar effects may occur in RVO-related macular edema. Moreover, it seems that ROCK inhibitor can also influence VEGF-related pathway, because CMT significantly decreased in our patients after combination therapy and remained at a plateau level after continuous injections of bevacizumab during follow up, whereas macular edema was refractory to previous anti-VEGF therapy in all patients. The interaction between VEGF and ROCK pathways during ischemic events (the point which can be concluded indirectly from our findings) has been investigated in experimental studies. It has been reported that VEGF stimulation could increase ROCK activity in retinal endothelial cells [8], and ROCK inhibitors could reduce VEGF expression (15) and suppress VEGF-induced endothelial cell properties [8]. Although suppression of both pathways leads to inhibition of neovascularization, it has been shown that ROCK inhibitors could induce intraretinal vascular growth leading to improvement in retinal perfusion and avascular area [8]. In a RVO murine model study, nonperfusion area and retinal edema was decreased and retinal blood flow was increased after intravitreal injection of ripasudil [16]. Furthermore, it was shown that in RVO, activity of ROCK was enhanced. Although there are some similarities between anti-VEGF drugs and Rock inhibitors in their function including antiangiogenesis, and antipermeability, noticeable differences also exist. In a review article with focus on vitreoretinal disorders, Yamaguchi et al. reported that inhibition of membrane contraction, antifibrosis, neuroprotection, and improvement of ischemia were distinctive effects of ROCK inhibitors from anti-VEGF drugs. They concluded that in some pathologic conditions including fibrosis in age related macular degeneration (ARMD), retinal ischemia, retinal neuropathy, and fibrovascular membrane contraction in diabetic retinopathy, ROCK inhibitors may be effective beyond VEGF inhibition [17]. Our study showed that RVO-related macular edema is another potential era for ROCK inhibitors.

In summary, this study revealed that Intravitreal ROCK inhibitors might break the resistance to anti-VEGF therapy and improve the RVO induced macular edema via affecting the VEGF-independent pathways. This study however had some limitations including the small sample size and the lack of a control group. More extended follow-up time would be needed to evaluate the long term effects. In addition, continued intravitreal injections of fasudil could manifest different results. Further randomized controlled trials with larger sample size and separate analysis of RVO subgroups are required.

## Data Availability

The datasets are available from corresponding author on reasonable request.

## Abbreviations

IVB: Intravitreal bevacizumab
RVO: Retinal vein occlusion
BCVA: Best corrected visual acuity
CMT: Central macular thickness
CRVO: Central retinal vein occlusion
BRVO: Branch retinal vein occlusion
VEGF: Vascular endothelial growth factor
IVF: Intravitreal fasudil
IOP: Intraocular pressure
DME: Diabetic macular edema
